# Infant Feeding and School Attainment in Five Cohorts from Low- and Middle-Income Countries

**DOI:** 10.1371/journal.pone.0071548

**Published:** 2013-08-20

**Authors:** Bernardo L. Horta, Abet Bas, Santosh K. Bhargava, Caroline H. D. Fall, Alan Feranil, Julia de Kadt, Reynaldo Martorell, Linda M. Richter, Aryeh D. Stein, Cesar G. Victora, The COHORTS group

**Affiliations:** 1 Post-Graduate Programme in Epidemiology, Universidade Federal de Pelotas, Pelotas, Brazil; 2 Office of Population Studies Foundation, University of San Carlos, Cebu, Philippines; 3 Sunderlal Jain Hospital, New Delhi, India; 4 MRC Lifecourse Epidemiology Unit, University of Southampton, Southampton, United Kingdom; 5 Developmental Pathways for Health Research Unit, University of the Witwatersrand, Johannesburg, South Africa; 6 Hubert Department of Global Health, Rollins School of Public Health, Emory University, Atlanta, Georgia, United States of America; 7 Human Sciences Research Council, Pretoria, South Africa; Universidade Federal do Acre (Federal University of Acre), Brazil

## Abstract

**Background:**

Performance in intelligence tests tends to be higher among individuals breastfed as infants, but little is known about the association between breastfeeding and achieved schooling. We assessed the association of infant feeding with school achievement in five cohorts from low- and middle-income countries. Unlike high-income country settings where most previous studies come from, breastfeeding is not positively associated with socioeconomic position in our cohorts, thus reducing the likelihood of a spurious positive association.

**Methodology and Principal Findings:**

Participants included 10,082 young adults from five birth cohorts (Brazil, India, Guatemala, the Philippines, and South Africa). The exposures variables were whether the subject was ever breastfed, total duration of breastfeeding, and age at introduction of complementary foods. We adjusted the estimates for age at follow up, sex, maternal age, smoking during pregnancy, birthweight and socioeconomic position at birth. The key outcome was the highest grade achieved at school. In unadjusted analyses, the association between ever breastfeeding and schooling was positive in Brazil, inverse in the Philippines, and null in South Africa; in adjusted analyses, these associations were attenuated. In Brazil, schooling was highest among individuals breastfed for 3–12 months whereas in the Philippines duration of breastfeeding was inversely associated with schooling; and null associations were observed in South Africa and Guatemala. These associations were attenuated in adjusted models. Late introduction of solid foods was associated with lower schooling achievement in Brazil and South Africa.

**Conclusion:**

Measures of breastfeeding are not consistently related to schooling achievement in contemporary cohorts of young adults in lower and middle-income countries.

## Introduction

In high-income countries, performance on intelligence tests tends to be positively associated with breastfeeding duration [1]. Most such studies are observational and may be affected by residual confounding and self-selection [2]. Cognition and performance on intelligence tests are positively associated with stimulation received by the child, [3], [4] and cognitive stimulation and emotional support tend to be higher among children who are breastfed [5]. Indeed, adjustment for characteristics of the home environment reduces the magnitude of the association between breastfeeding and performance on intelligence tests [5], [6]. Few studies on this topic are available from low and middle-income countries [7], [8].

Two randomized trials have explored this association. In a large cluster-randomized trial in Belarus, higher intelligence scores at the age of six years were observed in children born in hospitals allocated to the Baby-Friendly Hospital Initiative that included breastfeeding promotion and counseling. [9]. In another trial, preterm neonates were randomly assigned to receive breastmilk or formula in neonatal intensive care units; performance on intelligence tests in adolescence was higher among those who received breastmilk. [10]. These experimental results suggest that residual confounding does not fully account for the findings from observational studies. A postulated mechanism is the presence of long-chain polyunsaturated fatty acids (LC-PUFA; docosahexaenoic acid and arachidonic acid) in breast milk [11] [12].

Both in observational and randomized studies, the observed differences between the breastfeeding and comparison groups tends to be small, typically about three to five IQ points [13]. It is possible that such effects may not be reflected in long-term differences in “hard” outcomes, such as the number of years of schooling attained.

Based on data from a consortium of five long-term birth cohorts in low and middle-income countries, [14] we assessed the association between early feeding practices and long-term school achievement. Unlike studies from high-income populations, breastfeeding duration did not increase with socioeconomic position in our cohorts, so that one would not expect confounding to cause spurious positive associations [2].

## Methods

### Study populations

We analyzed data from the 5 birth cohorts in low- and middle-income countries that form the COHORTS collaboration, [14] including the 1982 Pelotas (Brazil) Birth Cohort, [15] the Institute of Nutrition of Central America and Panama Nutrition Trial Cohort (INTC; Guatemala), [16] the New Delhi (India) Birth Cohort Study, [17] the Cebu Longitudinal Health and Nutrition Survey (CLHNS; Cebu, Philippines), [18] and the Birth to Twenty (Bt20; South Africa) cohort [19].

The protocols of all studies were reviewed and approved by appropriate ethics review committees, and participants (or their parents, as appropriate) provided written consent. The following research ethics committees reviewed and approved the protocols: Brazil (Comitê de Ética em Pesquisa – Faculdade de Medicina – Universidade Federal de Pelotas); South Africa (University of the Witwatersrand Human Research Ethics Committee); Guatemala [Institutional Review Boards of the Institute of Nutrition of Central America and Panama (INCAP) and Emory University]; India (Ethics Committee of Sunder Lal Jain Hospital, Delhi); Philippines (UNC School of Public Health Institutional Review Board). All cohorts were population-based and recruited during gestation or at delivery. The Guatemala study was a cluster-randomized nutrition intervention trial carried out in four rural communities; intervention/comparison status was treated as a confounding variable in the analysis. Selected characteristics of the cohorts are presented in Table 1.

**Table 1:** Characteristics of the five cohorts studies and how infant-feeding data were collected.

Cohort	Design	Cohort inception (year)	Cohort description	How infant feeding data was collected	Last year of follow-up, and sample size
Pelotas birth cohort, Brazil	Prospective cohort	1982	Children born in the city's maternity hospital (>99% of all births) in 1982. All social classes included	Mothers were asked at 12, 20 and 48 months if they were breastfeeding and, if not, when they stopped. At 12 and 20 months, they were asked at what age other liquids and foods were added. Predominantly breastfeeding was defined as breast milk plus water or herbal teas only. Data from earlier visits were used preferentially	2005, 4297
INCTS, Guatemala	Community trial	1969–77	Intervention trial of high-energy and protein supplement in women and children <7 years in 1969 or born between 1969 and 1977 in four villages	Mothers were asked every 15 days, starting at birth, if they were breastfeeding. From the age of 15 months to 5 years, 24 hours dietary intake recall were gathered, every 3–6 months.	2004, 1571
New Delhi birth cohort study, India	Prospective cohort	1969–72	Children whose married mother lived in an area of Delhi. Primarily middle class sample	At 3, 6, 9, 12, 18, and 24 months mothers were asked about child diet.	1998–2002, 1583
CLHNS, Philippines	Prospective cohort	1983–84	Pregnant women living in 33 randomly selected neighborhood, 75% urban. All social classes included	At every 2 months from 0 to 24 months, a 24-h recall of all foods and liquids was performed.	2005, 2032
Birth-to-twenty, South Africa	Prospective cohort	1990	Babies born to pregnant women who lived in an area of Johannesburg. Predominantly poor, black sample	At 3, 6, 12, and 24 months, mothers were asked whether the child was breastfed; if not, when breastfeeding stopped; and when other milk, semi- solid or solid foods were introduced.	2009, 2225

### Breastfeeding

Information on breastfeeding duration and age at introduction of complementary foods were collected prospectively. Further details on the methods used to collect feeding information in each site are available elsewhere [20]. Three measures of infant feeding patterns are common across multiple cohorts and are used in the present analysis: (I) whether the subject was ever breastfed; (II) total duration of breastfeeding (not available for India); and (III) age at introduction of complementary foods (not available for Guatemala).

### Schooling

The highest grade of school successfully completed was collected in all five cohorts. In South Africa, 23.4% were still attending school at the time of follow-up, compared to 41.7% in Brazil and 0% in Guatemala, India, and the Philippines. For those subjects who were still attending school, we also gathered information on the highest grade successfully attained.

### Confounding variables

Age at follow up, sex, maternal age, smoking during pregnancy, birthweight and socioeconomic position at birth were included as potential confounding variables. Socioeconomic position was derived separately for each cohort using principal component analyses of household income and/or assets and services (Brazil, Guatemala, Philippines and South Africa), or the father's occupation (India), and was coded on a five-point scale ranging from 1 (lowest) to 5 (highest). In Brazil and South Africa we also adjusted for self-reported skin color. At cohort recruitment, the India, Brazil and South Africa cohorts were all urban and the Guatemalan cohort was all rural; in the mixed urban/rural Philippines cohort we controlled for urbanicity [21].

### Missing data and final analysis sample

The distribution of missing data according to feeding groups is available elsewhere [20]. In Brazil, the Philippines and South Africa the number of subjects with missing data on breastfeeding and achieved schooling was minimal. The analytic sample consisted of 10082 individuals.

### Data analysis

We used linear regression models for highest grade attained and Poisson regression with robust adjustment of the variance for completion of at least 12 years of school [22]. Statistical comparisons between groups were based on tests of heterogeneity and linear trend in the case of ordinal variables, and the one with the lower P-value was presented. We tested for heterogeneity across cohorts by fitting an interaction term. Analyses were carried out with StataSE version 12.

## Results

Table 2 describes the study populations. Mean ages of participants ranged from 17.7 years in South Africa to 30.2 years in Guatemala. Schooling attainment varied across sites, with the average highest grade attained at school ranging from 5.0 years in Guatemala, where only 6.4% completed secondary school, to 13.4 years in India where 87.6% did so.

**Table 2:** Selected characteristics of participants included in the analyses, by study site*.

	Brazil	Guatemala	India	Philippines	South Africa
Gender					
Male	52.1%	49.2%	58.0%	53.1%	48.1%
Age at follow-up, y*	22.7±0.4	30.2±2.4	29.2±1.3	21.2±0.9	17.7±0.3
Birthweight (g)					
<2500	7.2%	8.9%	23.5%	9.8%	11.0%
2500–2999	23.7%	31.6%	45.9%	35.1%	29.4%
3000–3499	37.6%	38.7%	25.5%	41.2%	40.1%
3500–3999	25.7%	17.0%	4.6%	12.7%	17.4%
≥4000	5.9%	3.8%	0.6%	1.2%	2.2%
Small for gestational age	14.4%	29.1%	42.2%	23.5%	14.7%
Socioeconomic status categories					
1 (poorest)	20.1%	24.0%	2.0%	22.8%	25.9%
2	50.2%	21.8%	10.8%	17.3%	20.1%
3	18.3%	20.8%	22.0%	20.1%	26.2%
4	5.7%	17.8%	49.1%	20.4%	17.9%
5 (least poor)	5.7%	15.6%	16.2%	19.3%	10.0%
Highest grade attained, y*	9.4±3.2	5.0±3.4	13.4±3.3	10.8±3.2	11.1±1.5
Completed ≥12 years of school	15.3%	6.4%	87.6%	34.2%	61.6%
Total	3847	730	1448	2059	1998

* Values are means ± SD or percent.

Breastfeeding incidence was greater than 99% in Guatemala and India, 95% in South Africa and Philippines, and 92% in Brazil (Table 3). Duration of breastfeeding was highly heterogeneous, with the proportion of children who were no longer breastfed at 6 months ranging from 2.7% (Guatemala) to 60.7% (Brazil); no data were available for India but prolonged breastfeeding was the norm. The median age at introduction of complementary foods ranged from less than 3 months in Brazil and South Africa, to 12 months in India (no data were available from Guatemala).

**Table 3:** Patterns of infant feeding of participants included in the analyses, by study cohort.

	Brazil	Guatemala	India	Philippines	South Africa
Ever breastfed	92.2%	99.7%	99.9%	94.9%	94.4%
Duration of any breastfeeding, mo					
≤1	31.2%	0.3%	N/A	11.0%	14.3%
>1–3	29.5%	0.6%		5.5%	16.4%
>3–6	14.3%	1.8%		6.6%	11.3%
>6–12	10.6%	14.9%		13.3%	14.9%
>12–18	4.1%	38.5%		27.5%	13.0%
>18–24	1.3%	28.8%		21.6%	10.1%
>24	8.9%	15.2%		14.5%	20.0%
Age at introduction of complementary foods, mo					
0–3	69.4%	N/ A	0.0 %	5.9%	57.9%
>3–6	28.2%		1.0%	81.7%	38.1%
>6–9	1.9%		8.3%	11.9%	3.4%
>9–12	0.5%		42.2%	0.5%	0.5%
>12–18	0.0%		36.5%	0.0%	0.1%
>18	0.0%		12.0%	0.0%	0.1%
Total	3847	730	1448	2059	1998

**Figure 1:**
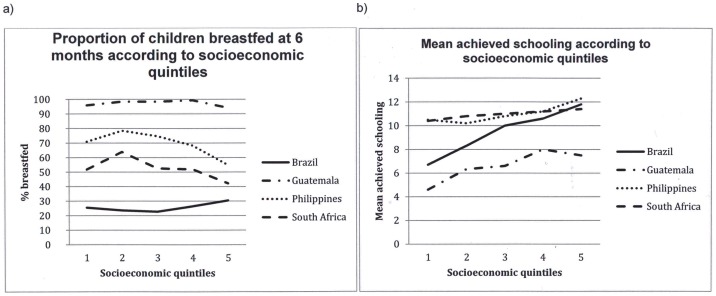
(a) Prevalence of breastfeeding at 6 months according to socioeconomic quintiles and (b) Mean achieved schooling according to socioeconomic quintiles.

Figure 1 shows the associations of breastfeeding and schooling with socioeconomic position. There was little variation in breastfeeding prevalence at six months by socioeconomic position in Guatemala (as almost all infants were still breastfed), and Brazil, whereas in the Philippines South Africa poorer women were more likely to breastfeed at 6 months. Socioeconomic status was positively associated with attained schooling in every site.

Tables 4 and 5 further describe the association of breastfeeding and schooling with the confounders. In Brazil, the proportion of children breastfed at 6 months was higher among those whose mother had low or high schooling, whereas in Guatemala and Philippines a negative association was observed. For maternal age, the direction of the association also changed according to study site, in Brazil the association was directly proportional, whereas in South Africa an inverse association was observed and no clear pattern was observed for Guatemala and Philippines. Birthweight was positively associated with prevalence of breastfeeding in Brazil and inversely related in The Philippines. Independent of study site, gender was not associated with prevalence of breastfeeding at 6 months(Table 4).

**Table 4:** Proportion of children breastfed at 6 months according to confounding variables, by study site.

	Brazil	Guatemala	India	Philippines	South Africa
Maternal schooling	<0.001#	0.02*		<0.001*	0.30#
None	37.6%	98.8%	N.A.	94.9%	46.2%
1–5 years	25.0%	95.9%		89.2%	68.0%
6–11 years	20.5%	94.1%		77.3%	58.1%
≥12 years	30.9%			41.2%	58.8%
Maternal age	<0.001*	0.99#		0.13#	0.03*
<20 years	21.5%	97.7%	N.A.	82.3%	63.9%
20–29 years	24.2%	97.3%		75.5%	57.1%
30–39 years	26.8%	97.2%		77.2%	57.2%
≥40 years	44.0%	97.6%		78.3%	40.6%
Birthweight (g)	<0.001*	0.24*		0.009#	0.08#
<2500	16.4%	95.8%	N.A.	71.4%	51.1%
2500–2999	22.6%	97.1%		74.7%	58.8%
3000–3499	24.8%	97.1%		79.9%	61.2%
3500–3999	28.4%	98.8%		77.8%	54.0%
≥4000	31.1%	100.0%		60.0%	57.9%
Gender	0.52	0.38		0.33	0.13
Male	24.5	97.9	N.A.	76.0	56.1
Female	25.4	96.8		77.9	59.8
Total	3847	730	1448	2059	1998

* test for linear trend.

# test for heterogeneity.

Achieved schooling was higher among those subjects whose mother had 12 or more years of schooling. The association of maternal age and birthweight with attained schooling was modified by study site. In Brazil, maternal age and birthweight were positively associated with achieved schooling, whereas in India the association was in the opposite direction. In the Philippines, highest grade at school was lower among those subjects whose mother had 40 years or more. In the majority of the studies site, achieved schooling was higher among females (Table 5).

**Table 5:** Mean achieved schooling according to confounding variables, by study site.

	Brazil	Guatemala	India	Philippines	South Africa
Maternal schooling	<0.001*	<0.001#	<0.001*	<0.001*	<0.001#
None	6.7	3.7	11.6	7.5	10.9
1–5 years	8.3	5.5	13.3	9.4	10.1
6–11 years	9.9	8.1	14.2	11.1	10.8
≥12 years	12.5	6.0	15.3	13.6	11.4
Maternal age	<0.001*	0.44#	0.002*	0.001#	0.58#
<20 years	8.5	4.9	13.8	10.4	11.0
20–29 years	9.4	4.8	13.4	11.0	11.1
30–39 years	9.7	4.4	13.0	10.9	11.0
≥40 years	9.6	4.6	11.7	9.7	10.8
Birthweight (g)	<0.001*	0.09*	<0.001#	0.20#	0.01#
<2500	8.6	4.6	13.0	10.4	10.9
2500–2999	8.9	5.1	13.4	10.9	10.9
3000–3499	9.4	4.8	13.9	10.9	11.2
3500–3999	9.8	5.4	14.4	11.1	11.1
≥4000	9.9	6.5	11.4	10.6	10.9
Gender	<0.001	<0.001	<0.001	<0.001	<0.001
Male	8.9	5.1	13.2	10.3	10.8
Female	9.8	4.3	13.9	11.4	11.3
Total	3847	730	1448	2059	1998

* test for linear trend.

# test for heterogeneity.

The association between breastfeeding incidence and achieved schooling was qualitatively heterogeneous across sites, but the interaction term was not statistically significant (p value  = 0.30) (Table 6). Among the three sites in which incidence was not universal, the association was null in South Africa, positive in Brazil and inverse in the Philippines. After controlling for confounding, the associations were markedly weakened and confidence intervals included unity.

**Table 6:** Highest grade achieved according to infant feeding in five birth cohorts.

	Regression coefficient (95% confidence interval) highest grade achieved at school				
	Brazil	Guatemala	India	Philippines	South Africa
***Unadjusted estimates***					
Ever breastfed	P = 0.03			P<0.001	P = 0.91
Yes	0.42 (0.05; 0.79)	&	N /A	−1.26 (1.89; −0.64)	0.02 (−0.26; 0.29)
No	0.0 (Reference)			0.0 (Reference)	0.0 (Reference)
Duration of any breastfeeding, mo	P<0.001#	P = 0.86#		P<0.001 *	P = 0.50
≤1.00	0.0 (Reference)		N /A	0.0 (Reference)	0.0 (Reference)
>1–3	0.25 (−0.01; 0.51)			−0.19 (−0.90; 0.52)	−0.06 (−0.32; 0.19)
>3–6	0.89 (0.57; 1.21)			−0.26 (−0.94; 0.41)	−0.10 (−0.38; 0.18)
>6–12	1.12 (0.77; 1.47)	0.0 (Reference)		−0.65 (−1.21; −0.09)	0.04 (−0.22; 0.30)
>12–18	0.47 (−0.05; 0.99)	0.13 (−0.66; 0.92)		−1.42 (−1.91; −0.94)	−0.02 (−0.29; 0.25)
>18–24	0.46 (−0.42; 1.34)	−0.11 (−0.93; 0.71)		−1.65 (−2.15; −1.14)	0.16 (−0.13; 0.45)
>24	−0.22 (−0.59; 0.16)	−0.15 (−1.09; 0.79)		−1.84 (−2.39; −1.30)	−0.12 (−0.37; 0.12)
Age at introduction of complementary foods, mo	P<0.001#		P = 0.15 *	P<0.001#	P = 0.06#
0–3	0.0 (Reference)	N /A		0.0 (Reference)	0.0 (Reference)
>3–6	−0.71 (−0.94; −0.49)			−0.50 (−1.10; 0.09)	0.09 (−0.04; 0.23)
>6–9	−1.57 (−2.30; −0.83)		0.0 (Reference)	−1.33 (−2.03; −0.62)	−0.27 (−0.64; 0.10)
>9–12	−1.47 (−2.85; −0.09)		−0.70 (−1.68; 0.28)	−0.51 (−2.56; 1.54)	0.88 (−0.88; 1.83)
>12–18			−0.82 (−1.82; 0.17)		
>18			−0.94 (−2.10; 0.23)		
***Adjusted estimates @***					
Ever breastfed	P = 0.65			P = 0.28	P = 0.41
Yes	−0.07 (−0.38; 0.24)	&	N /A	0.31 (−0.24; 0.85)	−0.08 (−0.41; 0.25)
No	0.0 (Reference)			0.0 (Reference)	0.0 (Reference)
Duration of any breastfeeding, mo	P = 0.07#	P = 0.69#		P = 0.71#	P = 0.47#
≤1.00	0.0 (Reference)		N /A	0.0 (Reference)	0.0 (Reference)
>1–3	0.03 (−0.18; 0.24)			0.16 (−0.45; 0.78)	−0.23 (−0.52; 0.05)
>3–6	0.36 (0.09; 0.62)			0.20 (−0.39; 0.78)	−0.10 (−0.41; 0.21)
>6–12	0.34 (0.04; 0.64)	0.0 (Reference)		0.40 (−0.09; 0.89)	−0.03 (−0.31; 0.26)
>12–18	0.18 (−0.26; 0.62)	0.35 (−0.53; 1.22)		0.11 (−0.33; 0.56)	−0.08 (−0.37; 0.21)
>18–24	0.22 (−0.52; 0.95)	0.39 (−0.52; 1.30)		0.06 (−0.40; 0.53)	0.09 (−0.21; 0.40)
>24	−0.01 (−0.32; 0.31)	−0.03 (−1.09; 1.03)		0.05 (−0.45; 0.55)	−0.02 (−0.29; 0.24)
Age at introduction of complementary foods, mo	P = 0.001 *		P = 0.62 *	P = 0.23 *	P = 0.15#
0–3	0.0 (Reference)	N /A		0.0 (Reference)	0.0 (Reference)
>3–6	−0.29 (−0.48; −0.10)			−0.15 (−0.67; 0.36)	0.05 (−0.09; 0.20)
>6–9	−0.43 (−1.05; 0.19)		0.0 (Reference)	−0.34 (−0.96; 0.27)	−0.08 (−0.47; 0.31)
>9–12	−0.47 (−1.64; 0.70)		0.08 (−0.88; 1.03)	−0.34 (−2.09; 1.41)	1.03 (0.10; 1.96)
>12–18			0.16 (−0.81; 1.13)		
>18			0.37 (−0.77; 1.52)		

& 2 subjects were never breastfed.

* test for linear trend.

# test for heterogeneity.

@ Adjusted for maternal socioeconomic status, maternal schooling, skin color (in Brazil and South Africa), age, smoking during pregnancy, birthweight, subject's age, sex, and urbanicity (in the Philippines).

For breastfeeding duration, heterogeneity across the four sites with data was also present (p for interaction <0.001). In Brazil, achieved schooling increased with breastfeeding duration up to 12 months, but decreased with longer durations. In the Philippines, duration was inversely related to schooling, while no association was observed in South Africa or Guatemala. The magnitude of all associations was reduced after controlling for confounding variables; in Brazil, the finding of greater schooling levels among subjects breastfed for 3 to 12 months persisted. In the Philippines, the effect of breastfeeding changed direction, in the adjusted analysis, and schooling was higher among those breastfed for 6 to 12 months, but not statistically significant. The test for heterogeneity across sites also decreased (p-value for interaction after adjustment  = 0.11).

Age at introduction of complementary foods was inversely related to school attainment in Brazil and the Philippines, but only the former remained significant after adjustment for confounders. We also stratified the analysis by sex (data not shown) and observed that for Guatemala (but not in the other sites) the association between duration of breastfeeding and achieved schooling was modified by sex (p-value for interaction  = 0.008). Among males, breastfeeding was positively related to school achievement, while for females the association was in the opposite direction.

In Brazil, the proportion of subjects who completed at least 12 years at school was higher among those who were ever breastfed (Table 7), while in the Philippines the opposite trend was observed (p-value for heterogeneity across sites <0.001). In the multivariate model, both associations disappeared.

**Table 7:** Completion of 12 or more years of schooling, according to infant feeding.

	Prevalence ratio (95% confidence interval) – completed at least 12 years of schooling				
	Brazil	Guatemala	India	Philippines	South Africa
***Unadjusted estimates***					
Ever breastfed	P = 0.001			P<0.001	P = 0.73
Yes	1.78 (1.23; 2.57)	&	N /A	0.53 (0.45; 0.63)	0.97 (0.84; 1.13)
No	1.0 (Reference)			1.0 (Reference)	1.0 (Reference)
Duration of any breastfeeding, mo	P<0.001#	P = 0.14#		P<0.001 *	P = 0.55#
≤1.00	1.0 (Reference)		N /A	1.0 (Reference)	1.0 (Reference)
>1–3	1.20 (0.97; 1.49)			0.90 (0.73; 1.11)	0.92 (0.80; 1.05)
>3–6	1.80 (1.44; 2.26)			0.74 (0.60; 0.93)	0.94 (0.81; 1.09)
>6–12	2.20 (1.75; 2.76)	1.0 (Reference)		0.63 (0.52; 0.77)	0.98 (0.85; 1.12)
>12–18	1.83 (1.30; 2.57)	0.39 (0.17; 0.87)		0.52 (0.44; 0.61)	0.97 (0.84; 1.11)
>18–24	1.02 (0.47; 2.19)	0.66 (0.31; 1.40)		0.49 (0.40; 0.59)	1.01 (0.87; 1.17)
>24	0.88 (0.62; 1.24)	0.53 (0.21; 1.39)		0.37 (0.29; 0.47)	0.89 (0.78; 1.02)
Age at introduction of complementary foods, mo	P<0.001#		P<0.001#	P<0.001#	P = 0.001#
0–3	1.0 (Reference)	N /A		1.0 (Reference)	1.0 (Reference)
>3–6	0.70 (0.58; 0.84)		1.0 (Reference)	0.79 (0.64; 0.94)	1.08 (1.00; 1.16)
>6–9	0.49 (0.22; 1.05)		0.94 (0.88; 1.01)	0.49 (0.36; 0.67)	0.88 (0.69; 1.12)
>9–12	0.87 (0.31; 2.49)		0.88 (0.84; 0.92)	0.90 (0.41; 1.97)	1.50 (1.18; 1.90)
>12–18			0.89 (0.85; 0.93)		
>18			0.85 (0.77; 0.93)		
**Adjusted estimates @**					
Ever breastfed	P = 0.29			P = 0.37	P = 0.59
Yes	1.20 (0.85; 1.69)	&	N /A	0.93 (0.80; 1.09)	0.95 (0.79; 1.14)
No	1.0 (Reference)			1.0 (Reference)	1.0 (Reference)
Duration of any breastfeeding, mo	P = 0.11#	P = 0.29#		P = 0.37#	P = 0.24#
≤1.00	1.0 (Reference)		N /A	1.0 (Reference)	1.0 (Reference)
>1–3	1.07 (0.89; 1.29)			1.03 (0.85; 1.24)	0.83 (0.70; 0.98)
>3–6	1.28 (1.05; 1.55)			0.92 (0.75; 1.13)	0.93 (0.78; 1.10)
>6–12	1.24 (1.02; 1.50)	1.0 (Reference)		0.94 (0.79; 1.13)	0.97 (0.83; 1.12)
>12–18	1.36 (1.00; 1.84)	0.46 (0.18; 1.22)		0.96 (0.81; 1.13)	0.94 (0.80; 1.09)
>18–24	0.87 (0.41; 1.85)	0.92 (0.38; 2.21)		0.96 (0.79; 1.16)	1.04 (0.89; 1.21)
>24	1.04 (0.76; 1.41)	0.53 (0.16; 1.76)		0.75 (0.59; 0.95)	0.97 (0.84; 1.12)
Age at introduction of complementary foods, mo	P = 0.57#		P = 0.80#	P = 0.29#	P = 0.004#
0–3	1.0 (Reference)	N /A		1.0 (Reference)	1.0 (Reference)
>3–6	0.92 (0.79; 1.08)		1.0 (Reference)	0.95 (0.78; 1.16)	1.06 (0.98; 1.15)
>6–9	1.06 (0.55; 2.03)		0.96 (0.86; 1.07)	0.78 (0.58; 1.04)	0.94 (0.75; 1.19)
>9–12	1.54 (0.63; 3.79)		0.95 (0.88; 1.04)	1.12 (0.49; 2.55)	1.58 (1.22; 2.06)
>12–18			0.99 (0.91; 1.07)		
>18			0.99 (0.88; 1.10)		

& 2 subjects were never breastfed.

* test for linear trend.

# test for heterogeneity.

@ Adjusted for maternal socioeconomic status, maternal schooling, skin color (in Brazil and South Africa), age, smoking during pregnancy, birthweight, subject's age, sex, and urbanicity (in the Philippines).

In Brazil, there was an increase in the proportion of subjects with 12 or more years of schooling with breastfeeding duration up to 18 months, followed by a decrease among those breastfed for a longer period. In contrast, data from the Philippines shows that breastfeeding duration was inversely related to this outcome. No association was observed in South Africa (where many were still in school) or Guatemala (where very few completed secondary school). The magnitude of the associations was strongly reduced after controlling for possible confounding variables. Later introduction of solid foods was associated with a lower likelihood of completing secondary school in Brazil, Guatemala and Philippines in the crude analyses, but the opposite trend was observed in South Africa. In adjusted models, only the association for South Africa remained significant, and those subjects with late introduction of foods were more likely to complete 12 or more years of schooling.

## Discussion

We assessed the associations between some measures of breastfeeding and complementary feeding with schooling achievement. Breastfeeding indicators did not show clear associations with schooling, with some heterogeneity across the five cohorts. Fewer than 10% of all infants were never breastfed in any of the cohorts, a much lower proportion than is observed in high-income countries [23]. In Guatemala and India, virtually all mothers started to breastfeed. Ever-breastfeeding tended to be positively associated with schooling in crude analyses in Brazil, negatively associated in the Philippines, and there was no association in South Africa. The findings from Brazil and Philippines disappeared after adjustment for confounders. Regarding breastfeeding duration, in Brazil the greatest levels of schooling were observed for subjects who were breastfed for 3–12 months, and in the Philippines there was a strong inverse association with longer breastfeeding associated with lower schooling attainment. There were no clear patterns in Guatemala or South Africa, and there were no data from India on breastfeeding duration. As for ever-breastfeeding, adjustment for confounding led to the associations in Brazil to virtually disappearing, and in the Philippines the association changed direction, schooling was higher among those breastfed for 6 to 12 months, but not statistically significant. Only two associations with significance levels below 0.05 were observed in the adjusted analyses, and both were related to the age of introduction of complementary foods. In Brazil, late introduction was associated with fewer years of schooling (but not with completing secondary school); in South Africa, the likelihood of completing secondary school was greater for those with late (nine months or older) introduction of complementary foods, but there was no corresponding difference in the number of years of schooling. Because of multiple associations being tested, these inconsistent results may well have been due to chance.

The overall lack of association between breastfeeding and schooling indicators is in contrast to consistent findings that performance in intelligence tests is positively associated with breastfeeding, both in observational and randomized studies [6], [8], [9]. These studies reporting positive associations were all carried out among children or adolescents, [9], [10], [13] and it is possible that the early advantage associated with breastfeeding does not translate into long-term educational achievement, which is affected also by other determinants. Furthermore, evidence suggests that the effect of early environmental characteristics on cognition may diminish with age [24].

Earlier findings from males in the Brazilian cohort at age 18 y [25] suggested that breastfeeding duration was positively related to the number of years of schooling completed, but at the time many were still in school. Furthermore, in the Philippines cohort, breastfed subjects showed improved performance in intelligence tests, but the effect was stronger at 8.5 than at 11.5 years of age, [7] which is in agreement with the notion that these effects may become attenuated over time.

In Brazil and Philippines, duration of breastfeeding was positively associated with achieved schooling among those subjects breastfed for up to 12 months, and in the pooled analysis, the confidence interval for those subjects breastfed from 3 to 12 months barely included the unity. But, we observed a decrease in the effect of breastfeeding among those breastfed for longer than 12 months. Evidence on the linearity of the association between breastfeeding and performance in intelligence tests or schooling are conflicting. Some studies have reported that duration of breastfeeding is positively related to performance in intelligence tests or achieved schooling, [5], [26]–[33] whereas other ones have observed a non-linear association, with a decrease in the effect of breastfeeding among those subjects who are breastfed for longer periods [8], [25], [34]–[37]. This heterogeneity among the studies may be due to differences in the categorization of breastfeeding duration, seven of the nine studies reporting a linear trend had >4 or >6 months as the highest category for breastfeeding duration, whereas the decrease in the effect of breastfeeding was observed among children breastfed for a longer time, ranging from 26+ weeks [34] to 24+ months [25], [35]. We also assessed the effect of the continuous variable duration of breastfeeding in months and the analyses carried out for each site were not statistically significant. We were not able to fit similar model for each site. In Brazil, we used a quadratic term to improve the fit of the model. In the Philippines, achieved schooling decreased 0.007 years (95% confidence interval: −0.02; 0.009) with the increase of one month in the duration of breastfeeding.

The present findings may also have been affected by other opportunities for achieving schooling outside the formal school system. For example, in Brazil, subjects who are behind in their schooling because of repeated failures can catch-up by taking special examinations and thus graduate from primary or secondary school at later ages, which means that a difference that would be observed during adolescence is no longer present among adults [25]. An individual who passed these examinations would be considered to have completed the corresponding number of school years. Furthermore, in South Africa, children start at school at 5–6 years of age with compulsory schooling to 16 years. For this reason, the distribution of attained schooling was homogeneous, with about 60% of cohort members having exactly 12 years of schooling and 93.6% having from 9 to 12 years. In Brazil and South Africa, schooling policies may therefore have contributed to an underestimation of any potential benefit of breastfeeding on school achievement.

Residual confounding, especially self-selection of mothers who breastfeed for longer periods of time, may have affected our findings. Furthermore, the small proportion of subjects who never breastfed is likely to be a highly selected sub sample. On the other hand, an earlier analysis of breastfeeding duration and intellectual performance in the 1993 Pelotas cohort and the British ALSPAC study took advantage of the different confounding structures in the two cohorts to explore causality [2]. Higher socioeconomic position was strongly associated with breastfeeding in ALSPAC, but there was little such patterning in Pelotas. In both studies breastfeeding was associated with higher IQ in childhood. In the present analyses, in the Philippines and South Africa the poor were more likely to breastfeed at 6 months, whereas in Brazil and Guatemala there were no clear social patterns. This may explain why many of the crude results in the Philippines go in opposite direction to those from other countries, particularly Brazil where the highest schooling is observed at 3–12 months of breastfeeding, at least in the unadjusted analyses. After controlling for confounding variables, the association in the Philippines changed direction and a small, but not statistically significant, benefit of breastfeeding on schooling seemed to be present. It could also be suggested that maternal leave and maternal employment could confound the association between breastfeeding duration and schooling. Because achieved schooling was independent of maternal employment, in the two sites with information on this variable, residual confounding due to maternal employment is unlikely. Indeed, adjustment for maternal employment scarcely changed the estimates.

To further address the issue of residual confounding we reanalyzed the data from the Philippines, including 11 additional confounders that were not included in the present analyses because they were not available from all sites (paternal presence in home, parity, alcohol during pregnancy, preterm status of child, mother reads, number of baths/week, dietary variety at age 2 years, household income, non-income-producing assets, electricity in home, and environmental hygiene score) [7]. Inclusion of these additional confounding variables in the analyses resulted in minimal changes in the regression coefficients shown in Table 6. For example, among those subjects who were breastfed from 6–12 month, the regression coefficient changed from 0.40 to 0.35, after controlling for the additional setting of confounding variables. Therefore, we retained the results adjusted for confounders that were available in all sites.

In summary, the early intellectual advantage of breastfed infants that is clearly documented in the literature is not paralleled by higher achieved schooling. This is likely to be explained by the fact that the relatively small increase in IQ points detected in childhood studies does not translate into greater schooling, when presumably also because there are so many other factors that determine school achievement.

## Supporting Information

File S1 List of the COHORTS group members.
(DOCX)
