# Supplementary material for: Infant Feeding and School Attainment in Five Cohorts from Low- and Middle-Income Countries
Source: PLoS One. 2013 Aug 20;8(8):e71548. doi: 10.1371/journal.pone.0071548 (PMC3748078; doi:10.1371/journal.pone.0071548)
Supplement: File S1 — List of the COHORTS group members. (DOCX) [file pone.0071548.s001.docx]

APPENDIX

The COHORTS group members are as follows: Pedro C. Hallal, Fernando C. Barros, Denise P. Gigante (Universidade Federal de Pelotas, Brazil); Manuel Ramirez-Zea (Institute of Nutrition of Central America and Panama, Guatemala City, Guatemala); Harshpal Singh Sachdev (SitaramBhartia Institute of Science and Research, New Delhi, India); Linda Adair (University of North Carolina at Chapel Hill, Chapel Hill, North Carolina); Judith Borja (Office of Population Studies, University of San Carlos, Cebu City, Philippines); Darren Dahly (University of Leeds, Leeds, U.K.); and Chris Desmond, Carren Ginsburg, Lisa Mickelesfield and Shane Norris (Developmental Pathways for Health Research Unit, Department of Paediatrics, Faculty of Health Sciences, University of the Witwatersrand, Johannesburg, South Africa); and Alan Stein (Department of Psychiatry, University of Oxford); and Clive Osmond (MRC Epidemiology Resource Centre, University of Southampton, Southampton, UK).
